# Roles of GP33, a guinea pig cytomegalovirus-encoded G protein-coupled receptor homolog, in cellular signaling, viral growth and inflammation *in vitro* and *in vivo*

**DOI:** 10.1371/journal.ppat.1007487

**Published:** 2018-12-20

**Authors:** Miei Takeda, Shinji Watanabe, Harutaka Katano, Kazuma Noguchi, Yuko Sato, Sayaka Kojima, Takuya Miura, Ryuichi Majima, Souichi Yamada, Naoki Inoue

**Affiliations:** 1 Laboratory of Microbiology and Immunology, Gifu Pharmaceutical University, Gifu, Japan; 2 Department of Pathology, National Institute of Infectious Diseases, Tokyo, Japan; 3 Department of Virology I, National Institute of Infectious Diseases, Tokyo, Japan; Oregon Health and Science University, UNITED STATES

## Abstract

Cytomegaloviruses (CMVs) encode cellular homologs to evade host immune functions. In this study, we analyzed the roles of GP33, a guinea pig CMV (GPCMV)-encoded G protein-coupled receptor (GPCR) homolog, in cellular signaling, viral growth and pathogenesis. The cDNA structure of GP33 was determined by RACE. The effects of GP33 on some signaling pathways were analyzed in transient transfection assays. The redET two-step recombination system for a BAC containing the GPCMV genome was used to construct a mutant GPCMV containing an early stop codon in the GP33 gene (Δ33) and a rescued GPCMV (r33). We found the following: 1) GP33 activated the CRE- and NFAT-, but not the NFκB-mediated signaling pathway. 2) GP33 was dispensable for infection in tissue cultures and in normal animals. 3) In pregnant animals, viral loads of r33 in the livers, lungs, spleens, and placentas at 6 days post-infection were higher than those of Δ33, although the viruses were cleared by 3 weeks post-infection. 4) The presence of GP33 was associated with frequent lesions, including alveolar hemorrhage in the lungs, and inflammation in the lungs, livers, and spleens of the dams. Our findings suggest that GP33 has critical roles in the pathogenesis of GPCMV during pregnancy. We hypothesize that GP33-mediated signaling activates cytokine secretion from the infected cells, which results in inflammation in some of the maternal organs and the placentas. Alternatively, GP33 may facilitate transient inflammation that is induced by the chemokine network specific to the pregnancy.

## Author summary

Cytomegalovirus (CMV) is a major pathogen that causes congenital diseases, including birth defects and developmental abnormalities in newborns. Better understanding of the immune evasion mechanisms may open the way to the development of new types of live attenuated vaccines for congenital CMV infection. In contrast to murine and rat CMVs, guinea pig CMV (GPCMV) causes infection *in utero*, which makes GPCMV animal models a useful tool for understanding the pathogenesis of congenital infection and evaluation of vaccine strategies. By constructing a GPCMV mutant lacking GP33, a viral G protein-coupled receptor homolog, this study found that GP33 was involved in the induction of significant inflammatory responses in pregnant but not in normal animals. As GP33 activated the NFAT- and CRE-, but not the NFκB-signal pathway, it is plausible that GP33 enhanced cytokine expression, which results in pathogenic outcomes in the maternal organs and placentas.

## Introduction

Congenital infection of human cytomegalovirus (HCMV) occurs in 0.2–1% of all births and causes birth defects and developmental abnormalities, including neurological sequela [1–4]. In contrast to murine CMV (MCMV) and rat CMV (RCMV), guinea pig CMV (GPCMV) causes infection *in utero* and pathogenic outcomes similar to those caused by HCMV, which makes GPCMV animal models useful for studies on congenital CMV diseases [5–8].

Virus-encoded G protein-coupled receptor homologs (vGPCRs) are characteristic of beta- and gamma-herpesviruses and are considered potential therapeutic targets. HCMV encodes four vGPCRs: UL33 and UL78 are conserved in all beta-herpesviruses, including MCMV, RCMV and GPCMV, whereas US28/US27 homologs are restricted to primate beta-herpesviruses. Although US28 and UL78 are expressed early after infection, US27 and UL33 are expressed during the late phase of infection [9]. It is well known that stimulation of GPCR activates heterotrimeric G proteins, which are composed of α, β, and γ subunits, and dissociates them into α and βγ subunits, resulting in production of second messengers for transcriptional gene regulation. Among the Gα family, Gαs stimulates cyclic AMP (cAMP) production, resulting in cAMP response element (CRE) activation, while Gαi inhibits cAMP production. Gαq stimulates phospholipase C (PLC), resulting in activation of protein kinase C (PKC) and nuclear factor of activated T-cells (NFAT). GPCRs associate with G proteins through a DRY (Asp-Arg-Tyr) motif located at the cytosolic end of the third membrane-spanning segment, and the motif plays an important role in the conformational transition between active and inactive states of GPCRs [10]. Although UL33 contains the conserved DRY motif, it activates PLC, p38, and CRE binding protein (CREB) constitutively [11]. A structural study on US28 suggests that the DRY motif and its immediate environment affect constitutive activity of viral GPCRs [12]. M33, an MCMV homolog of UL33, displays ligand-independent, constitutive signaling through the Gq/PLC pathway [13]. M33 also activates CREB in a ligand-independent, constitutive manner. M33 contains a NRY motif at the amino acid (aa) position 130–132 in place of the DRY motif, and the alteration R131A but not N130D in the motif abolished the constitutive signaling [14]. M33 and R33, but not UL33, activate the NFκB pathway. This activation by M33 is G protein-independent, but that by R33 is G protein-dependent [15,16]. Functional similarities in signaling activities between M33 and US28, including activation of the NFκB pathway and vascular smooth muscle cell migration, have been reported [13,17]. UL33 and UL78 form heterodimers with human CC chemokine receptor 5 (CCR5). The heterodimerization of UL33 impairs the CC chemokine ligand 5 (CCL5)-induced internalization of CCR5 and the CCR5-mediated cell migration and HIV entry [18].

The roles of MCMV and RCMV vGPCRs *in vivo* have been studied intensively. After CMV infection, acute-phase replication in the primary organs is followed by dissemination to the secondary sites, such as the salivary glands, where the virus may replicate for several days, affording an opportunity for horizontal viral spread. The lack of M33-dependent signaling decreased viral loads in the salivary glands [14,19]. A recombinant MCMV expressing US28 partially complemented the defect due to the lack of M33 in BALB/c mice infected intraperitoneally (i.p.) [20]. A recent study demonstrated that M33 is required for viral replication in the salivary glands in NOD/scid-/infg- (NSG) mice infected i.p. [21]. Intranasal (i.n.) administration of MCMV lacking M33 also demonstrated that transmission from the lymph nodes (LNs) to the lungs requires M33 functions [22].

As there are few studies on GPCMV GP33 and as the GPCMV animal model can address the question of whether GP33 plays any role in congenital infection, this study sought to characterize the roles of GP33 in cellular signaling and viral pathogenesis, particularly under pregnant conditions.

## Results

### cDNA structure of the GP33 gene and localization of its product

**Fig 1:**
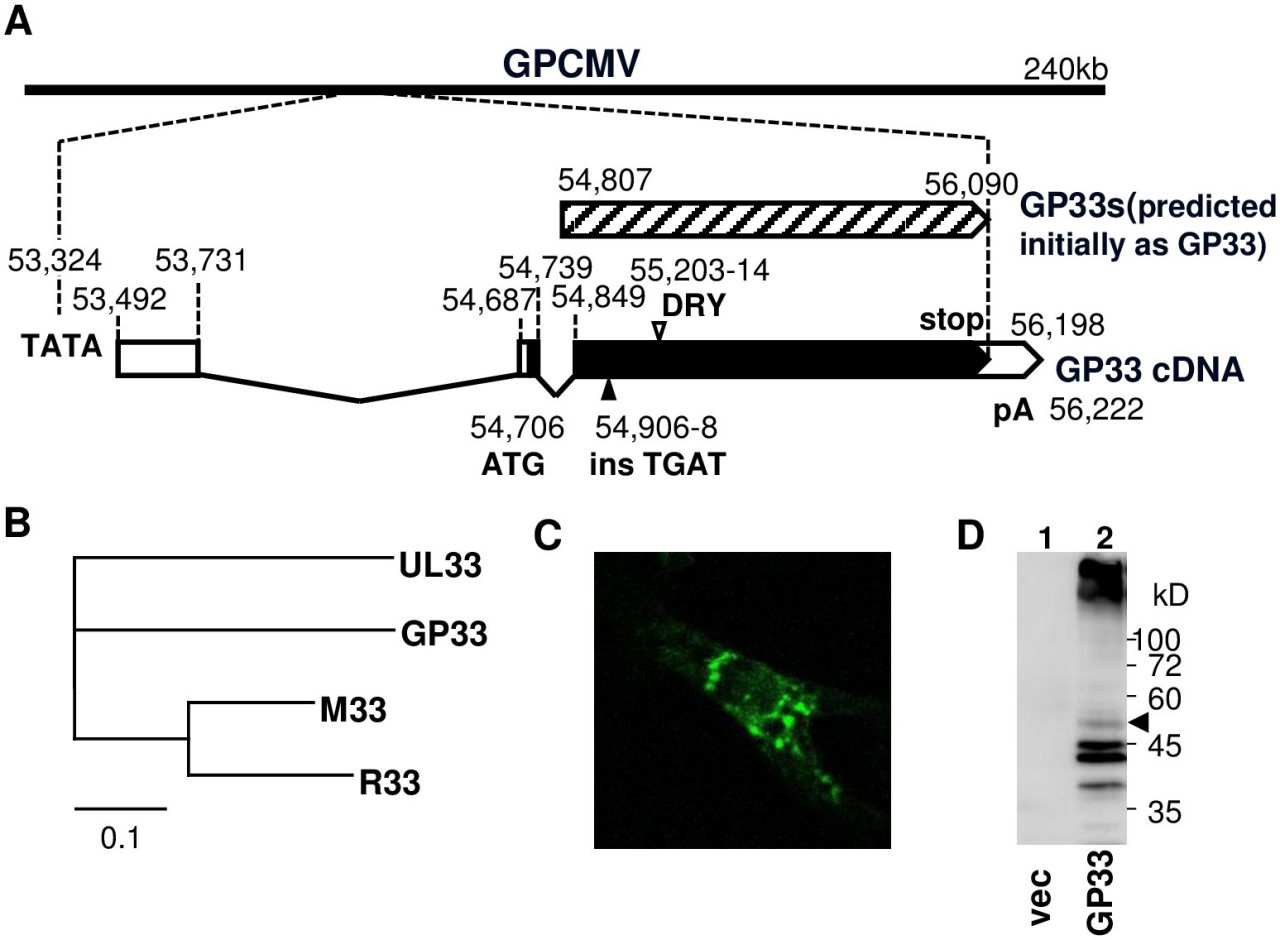
cDNA structure and expression of GP33. **(A)** The GP33 cDNA structure determined by RACE analyses is depicted with a TATA box and a poly A signal (pA). The numbers in the GPCMV genome positions are based on Kanai et al. [33]. The ORF predicted initially as GP33 (hatched box; GP33s) and the ORF determined by RACE (closed boxes; GP33) are shown. An open arrowhead indicates the location of the DRY motif. “ins TGAT” is the mutation containing a stop codon and a 1-bp insertion to generate the GP33-defective virus, Δ33. **(B)** Multiple sequence alignment of GP33 homologs, HCMV UL33, MCMV M33, and RCMV R33, was performed by ClustalW analysis (DDBJ, ver. 2.1) with the default parameters except for GAP OPEN 100, and the obtained bootstrapped tree is depicted by TreeViewX software. **(C)** Localization of GP33-EGFP fusion protein in COS-7 cells transiently transfected with pEGFP-GP33. **(D)** Lysates of cells transfected with pcDNA3 empty vector (lane 1) or pcDNA-GP33F (lane 2) were separated on SDS-10% polyacrylamide gel. Proteins in the gel were transferred to polyvinylidene difluoride membrane (Millipore), and reacted with monoclonal anti-FLAG tag antibody followed by peroxidase-conjugated anti-mouse IgG. An arrowhead indicates the expected full-length GP33 band.

RNA samples were prepared from guinea pig lung fibroblasts (GPL) at 3 days after infection with GPCMV. The cDNA structure of the GP33 gene was determined by RACE analyses using the primers listed in S1 Table. The GP33 gene consists of three exons that encode a 424 amino acid (aa) product (Fig 1A) and contain the conserved DRY motif at the aa positions 130–132. Homologies of the GP33 aa sequence with GP33 homologs of other herpesviruses are more than 85% with similar distances from GP33 to UL33 and to M33 (Fig 1B).

A plasmid encoding GP33 fused with EGFP at the carboxyl side was constructed. Immunofluorescence assay (IFA) of the cells that were transfected with the plasmid and fixed with paraformaldehyde demonstrated that GP33 was localized mainly in some vesicles in the cytoplasm and slightly in the plasma membrane (Fig 1C), as shown in cells expressing UL33 [11,23]. Plasmids encoding the GP33 ORF without and with a FLAG-tag at the carboxyl end were also constructed. Immunoblotting of the transfected cell lysate with anti-FLAG antibodies demonstrated that GP33 formed a ~50 kDa band but was also heavily aggregated (Fig 1D).

### Signal pathways induced by GP33

**Fig 2:**
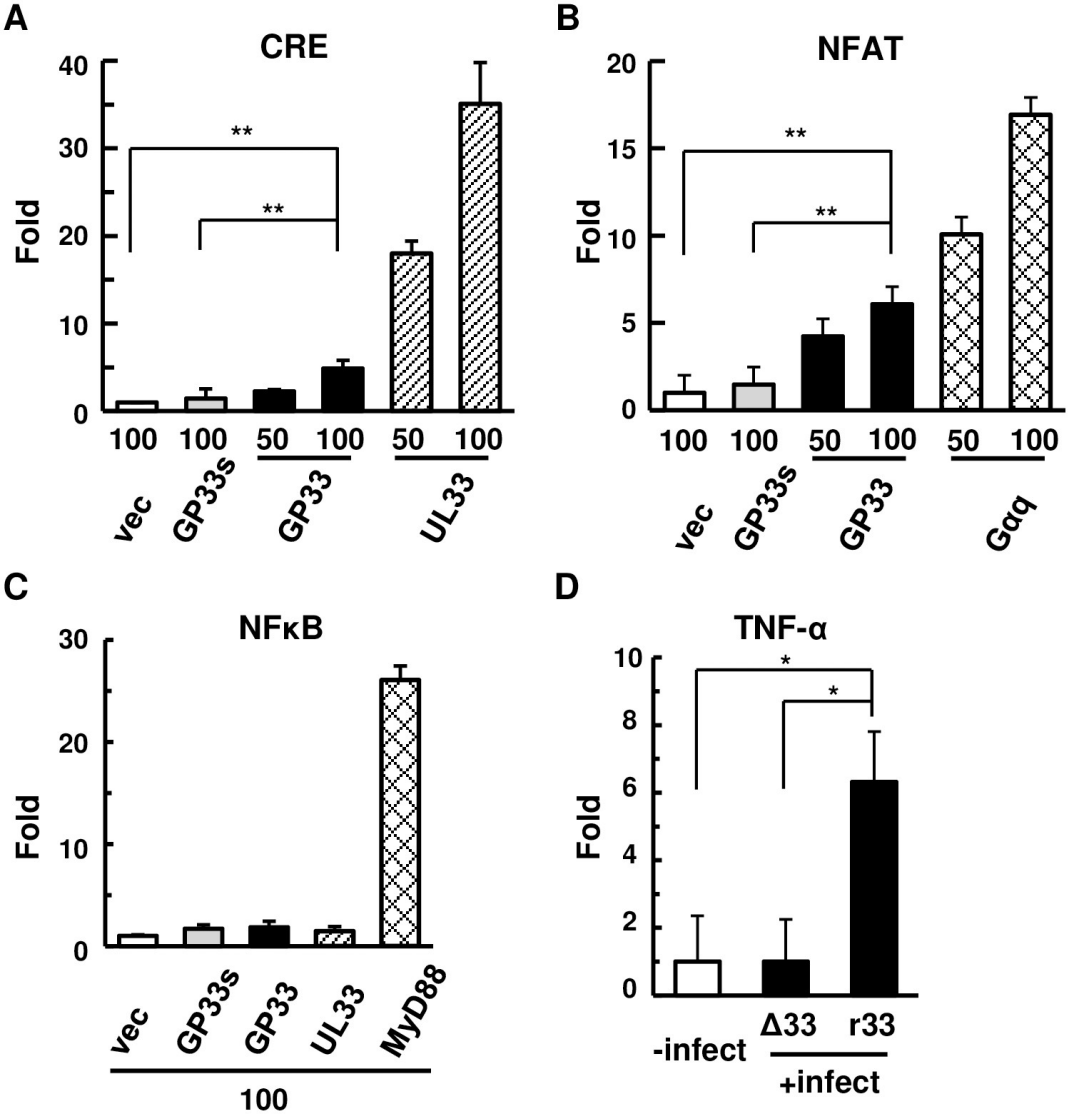
Signal pathways activated by GP33. **(A-C)** COS-7 cells were transfected with 50 ng of a reporter plasmid, pCRE-luc (A), pNFAT-luc (B) or pNFκB-luc (C), 0.5 ng of pRL-EF1α, and the indicated amount (ng) of an empty vector (vec) or an effector plasmid. pcDNA-GP33s (GP33s), -GP33 (GP33), -UL33 (UL33), pF4A-CMV-GαqQ209L (Gαq), and a plasmid expressing FLAG-MyD88 (MyD88) were used as the effector and control plasmids. Means and standard error of means (SEM) of fold induction obtained in triplicated wells using the relative luciferase activity of the cells transfected with an empty vector (vec) as a standard are shown. **: p<0.01. **(D)** TNF-α mRNA levels in uninfected GPL cells, or those infected with Δ33 or r33 at an MOI of 1. RNA samples were prepared from GPL cells at 1 day p.i. The amounts of TNF-α cDNA relative to those of β-actin were measured using real-time RT-PCR assay. Means and SEM of the fold increases obtained in triplicated wells using the relative TNF-α level in the uninfected cells as a standard are shown. *: p<0.05.

Next, transient transfection of a CRE reporter plasmid along with GP33s lacking exons 1 and 2 of GP33, GP33 cDNA, and HCMV UL33, respectively, demonstrated that GP33 weakly activated CRE and that an 11-aa sequence from the amino-terminal end of GP33, which is absent in GP33s, was important for this CRE activation (Fig 2A), probably due to a difference in the efficiency of correct subcellular trafficking [11,14] or of glycosylation at an NXT motif just before the cysteine residue of GP33, which is conserved among the UL33 homologs, near the amino-terminal region [24]. In addition, the NFAT- but not the NFκB-signal pathway was activated by GP33 (Fig 2B and 2C). As M33 but not UL33 activates the NFκB pathway [13] and as the homology distances of GP33 to UL33 and M33 are similar, GP33 may function in manner more closely resembling that of UL33.

### GP33-induced increase of tumor necrosis factor alpha (TNF-α) expression

To elucidate the roles of GP33 in the viral dissemination and pathogenesis, GFP-expressing GPCMV with a stop codon mutation in the GP33 ORF (Δ33) and that with the rescued wild-type GP33 ORF (r33) were generated using the two-step redET recombination method. Rawlinson and his colleagues demonstrated upregulation of some pro-inflammatory cytokines in the placenta and amniotic fluid of congenitally infected fetuses in human as well as increase of MCP-1 and TNF-α expression in HCMV-infected ex vivo placental explant [25,26]. In addition to those findings, since it is well known that NFAT signaling activates TNF-α expression in some cell types and that NFAT can interact with several transcription factors, including those downstream of GPCR signaling, for synergistic activation (reviewed in [27–29]), we analyzed the effect of GP33 on TNF-α expression. As shown in Fig 2D, real-time reverse transcription (RT) PCR assays demonstrated that TNF-α mRNA level in r33-infected GPL cells was higher than that in Δ33-infected GPL cells (p<0.05).

### GP33 is dispensable for viral growth in tissue cultures

**Fig 3:**
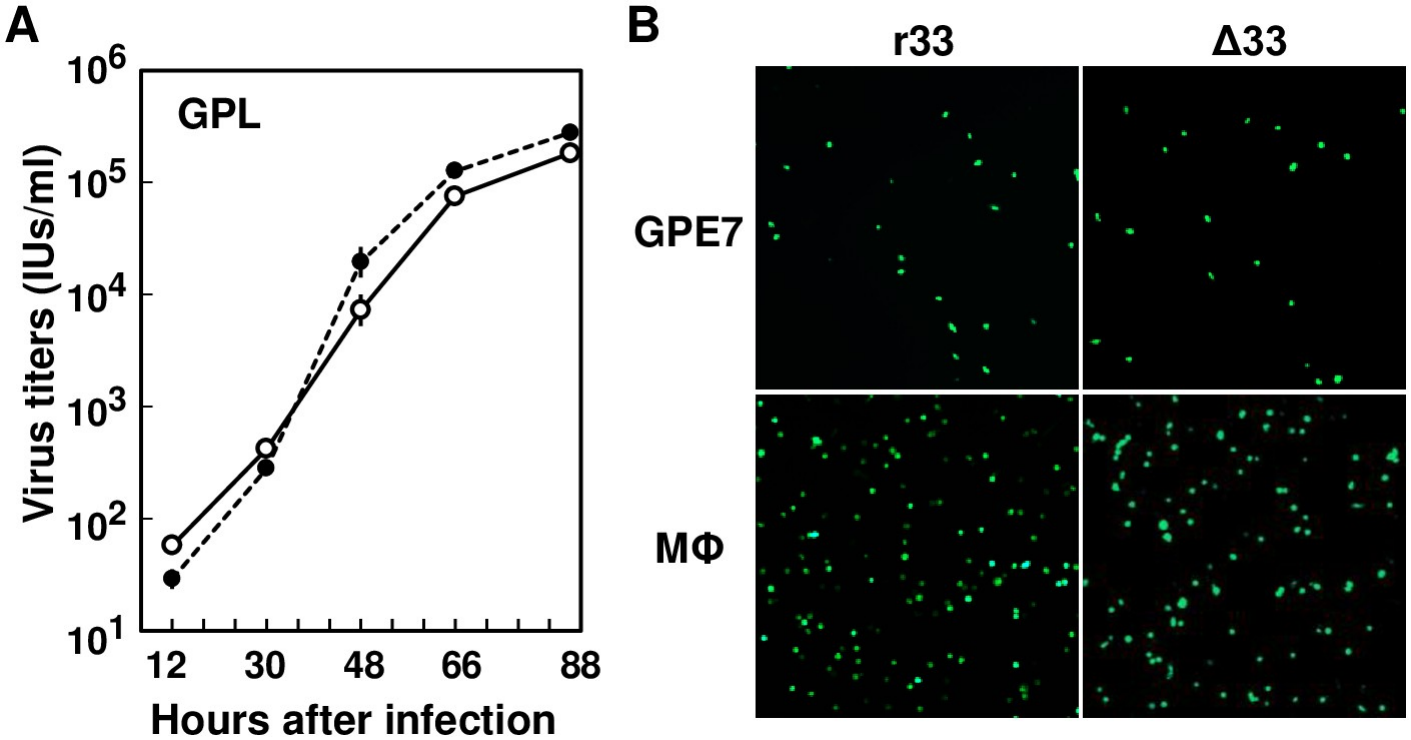
Viral infection *in vitro*. **(A)** GPL cells in 48-well plates were infected with GPCMV r33 and Δ33 at an MOI of 0.05, and culture supernatants were recovered and frozen at the indicated hours after infection. The viral titers (IUs) were determined by counting GFP-positive cell foci at 2 days after infection. Means and SEMs of the virus stocks prepared in triplicated wells are plotted for r33 (open circles) and Δ33 (closed circles). **(B)** Representative images of GFP-positive cells after infection of GPE-7 cells and macrophages (Mϕ) with r33 or Δ33 at an MOI of 10 (the virus titers were determined in GPL cells) at 24 hrs and 36 hrs p.i., respectively.

In GPL cells, GPCMV r33 and Δ33 were found to grow to a similar extent (Fig 3A). In addition, both viruses infected guinea pig epithelial cell clone GPE-7 as well as macrophages prepared by TPA-treatment of splenic monocytes at a similar level (means and standard errors of means (SEMs) of GFP+ cells/well were r33 = 1,010 ± 135 vs. Δ33 = 1,206 ± 107 and r33 = 2,406 ± 388 vs. Δ33 = 1,741 ± 114, respectively) (Fig 3B). These results indicate that GP33 is dispensable for infection and growth *in vitro*.

### GP33 had no apparent effects on viral growth in normal animals

**Fig 4:**
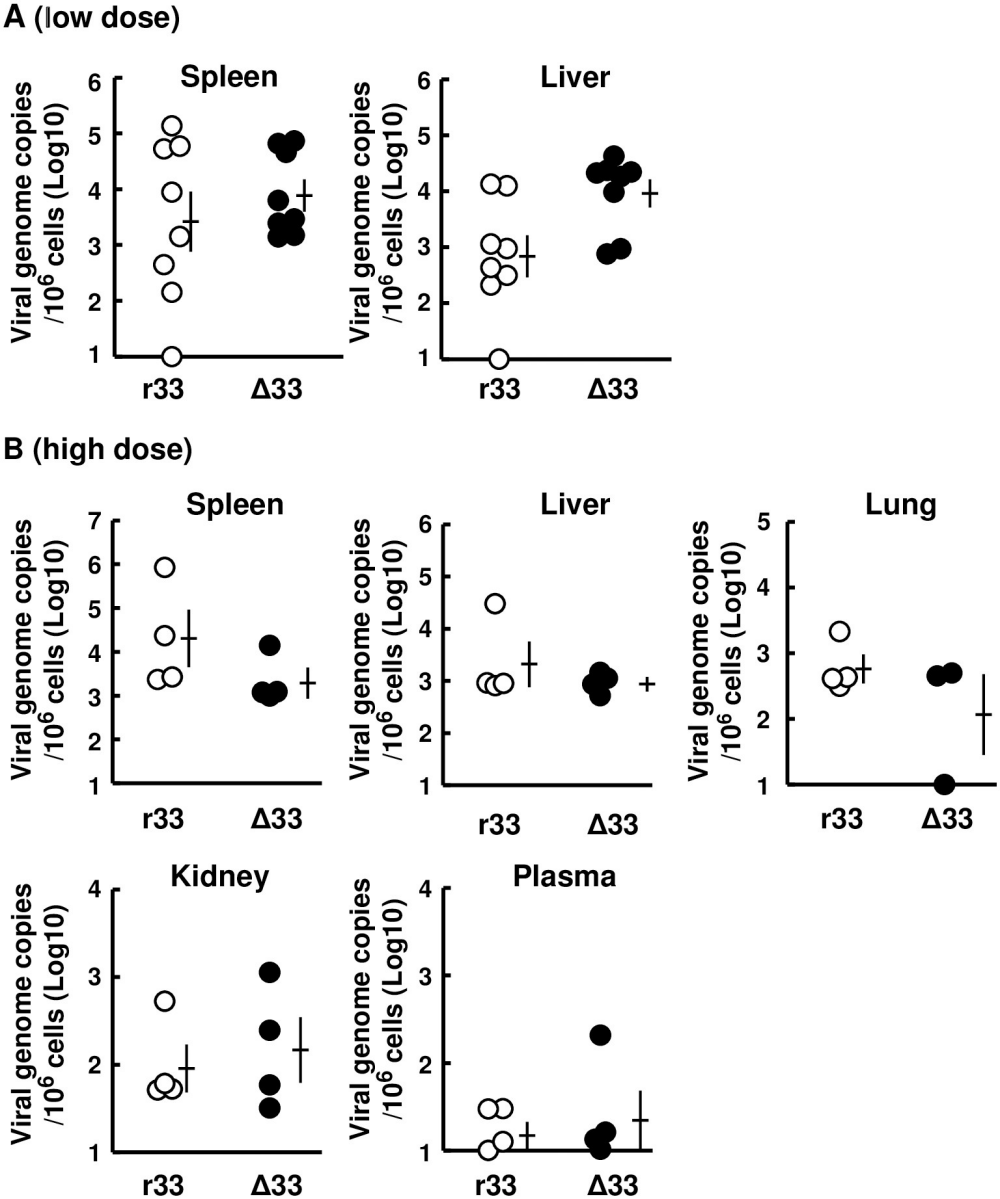
Viral loads in organs after infection of normal animals. Female 4-week-old guinea pigs (Hartley) were infected i.p. with r33 (open circles) or Δ33 (closed circles) at a low dose (n = 8 each, 1x10^6^ IUs/animal) **(A)** and at a high dose (n = 4 each, 2x10^7^ IUs/animal) **(B)**. GPCMV genome copies per 10^6^ cells of the indicated organs obtained at 1 week after the infection are plotted. Means and SEMs are shown.

Female 5-week old normal guinea pigs (Hartley) were infected i.p. with 1x10^6^ or 2x10^7^ infectious units (IUs)/animal of GPCMV (r33 or Δ33) and sacrificed at 1 week post-infection (p.i.). There were no apparent abnormalities in the normal animals infected with r33 or with Δ33. No significant differences in the appearance of any organs were observed irrespective of the dose, low (1x10^6^ IUs/animal) or high (2x10^7^ IUs/animal), and there were no differences in viral loads in their livers, spleens, lungs, kidneys, or plasma specimens (Fig 4).

### The defect in GP33 decreased viral loads in dams at 6 days p.i

**Fig 5:**
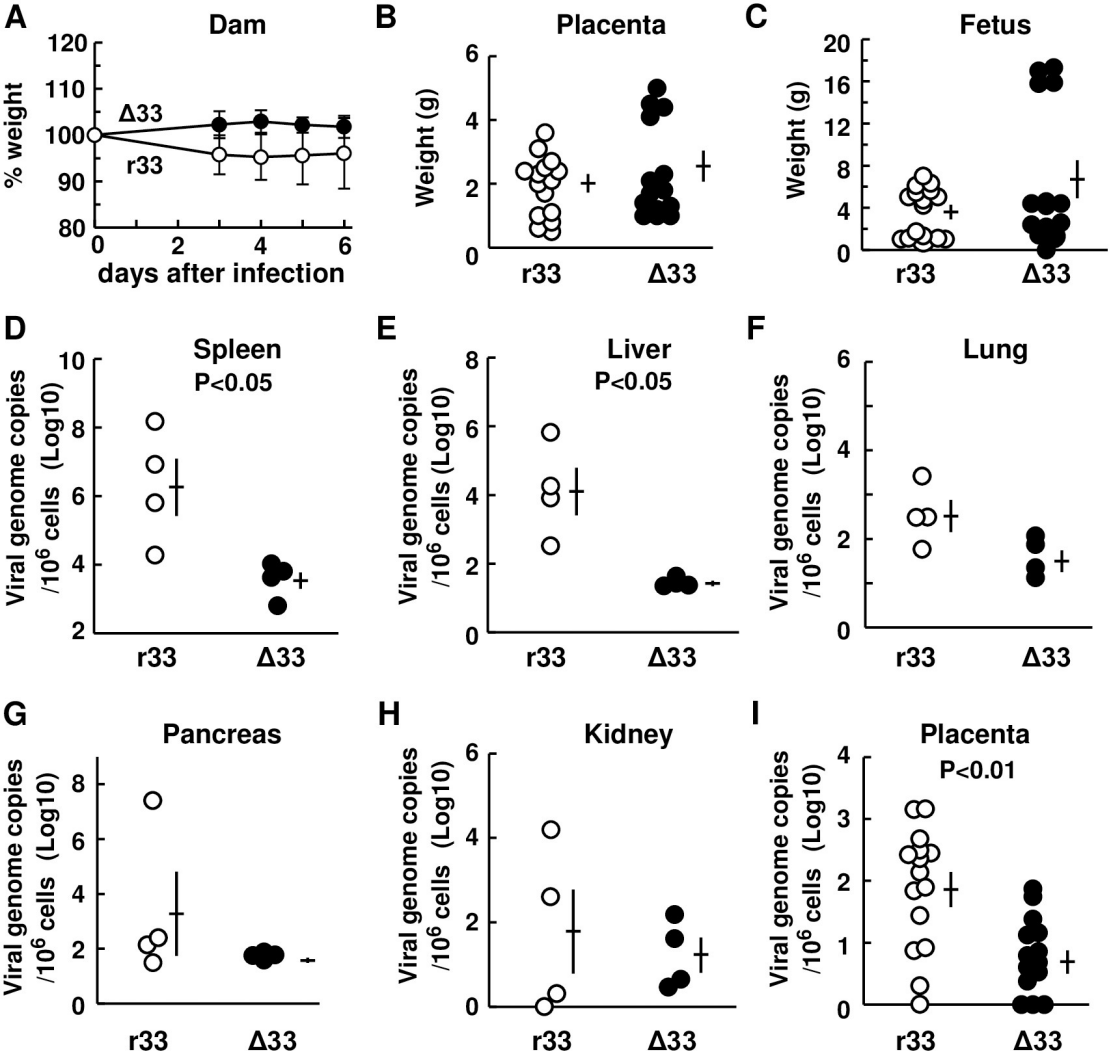
Viral loads in organs at 6 days after infection of dams at 4 weeks’ GA. Pregnant guinea pigs (Hartley) at 4 weeks’ GA were infected s.c. with GPCMV r33 or Δ33 (n = 4 each, 2x10^7^ IUs/animal) and sacrificed at 6 days p.i. to collect specimens. **(A)** Relative body weights of dams infected with r33 (open circles) or Δ33 (closed circles) (n = 4 each) are shown using the body weight of each animal at the time of GPCMV infection as a 100% control. Comparison of the weights of placentas **(B)** and fetuses **(C)** from dams infected with r33 (open circles) or Δ33 (closed circles). Each circle indicates one placenta or fetus. Means and SEMs are shown. **(D-H)** GPCMV genome copies per 10^6^ cells of the indicated organs from the animals infected with r33 (open circles) or Δ33 (closed circles). Means and SEMs are shown. **(I)** GPCMV genome copies per 10^6^ cells of the placentas obtained from the dams described above.

As it appears that GP33 is not involved in disease pathogenesis in normal animals, dams (Hartley) at 4 weeks’ gestational age (GA) were infected subcutaneously (s.c.) with GPCMV r33 or with Δ33 (n = 4 each; 1.2 x 10^7^ IUs/animal) and sacrificed at 6 days p.i. to see whether GP33 has any roles in the pathogenesis of congenital CMV diseases. There were no statistical differences in the body weight of r33- and Δ33-infected dams (Fig 5A) or in the weight of the placentas and fetuses from r33- and Δ33-infected dams (Fig 5B and 5C). However, viral loads in the spleens and livers of the r33-infected dams were significantly higher (p<0.05) than those of the Δ33-infected dams, although those in lungs, pancreas, and kidneys did not reach statistical significance (Fig 5D–5H). In addition, viral loads in the placentas of the r33-infected dams were significantly higher (p<0.01) than those of the Δ33-infected dams (Fig 5I). Due to the small size of the infected fetuses at 5 weeks’ GA, collection of the fetal organs from most fetuses was impossible.

### Inflammation in the maternal organs at 6 days p.i

**Fig 6:**
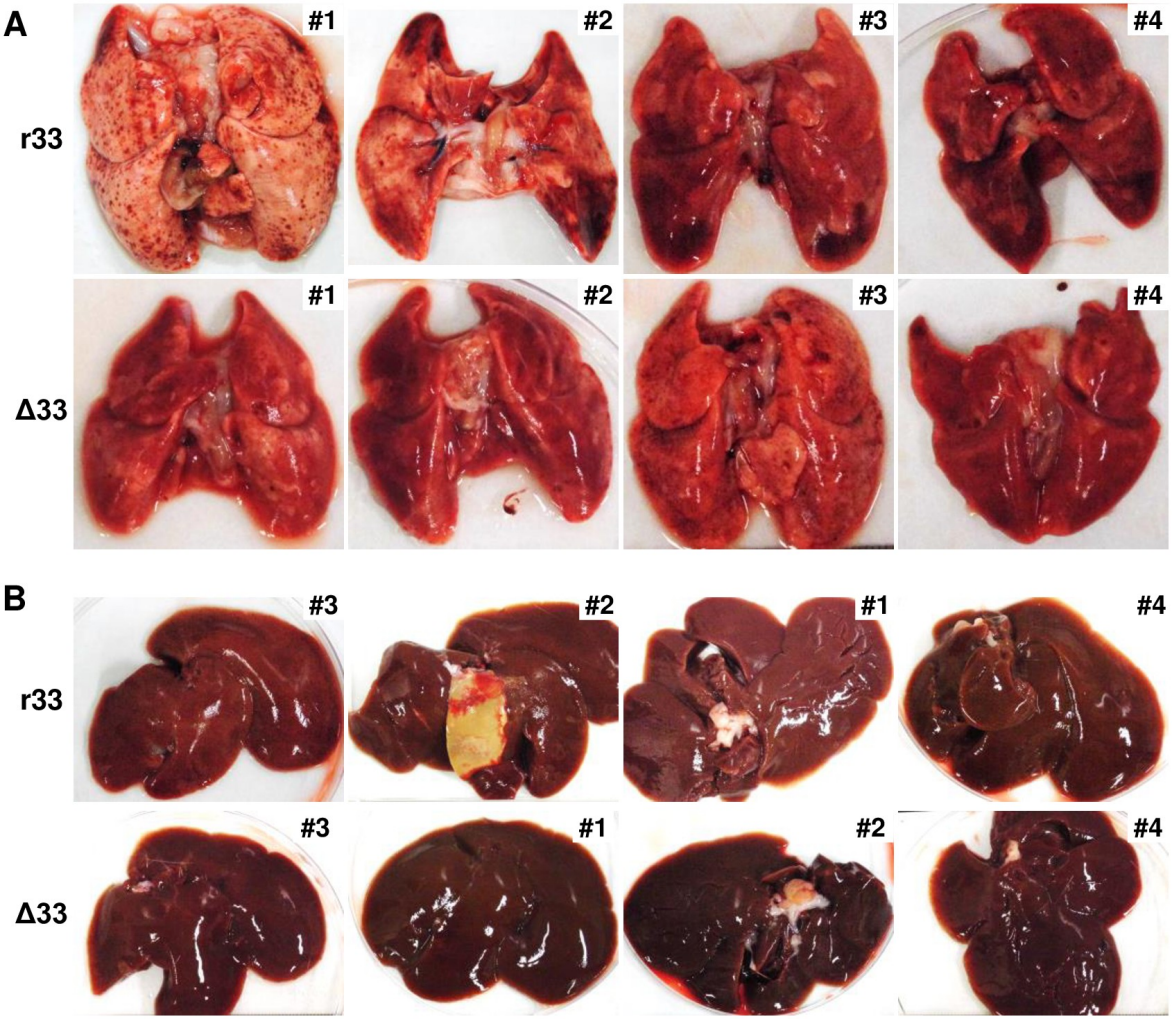
Appearance of the organs obtained from infected dams at 6 days p.i. Images of the lungs **(A)** and livers **(B)** obtained from the dams described in the legend for Fig 5 are shown in the order of abnormalities in appearance from most severe to mildest.

**Fig 7:**
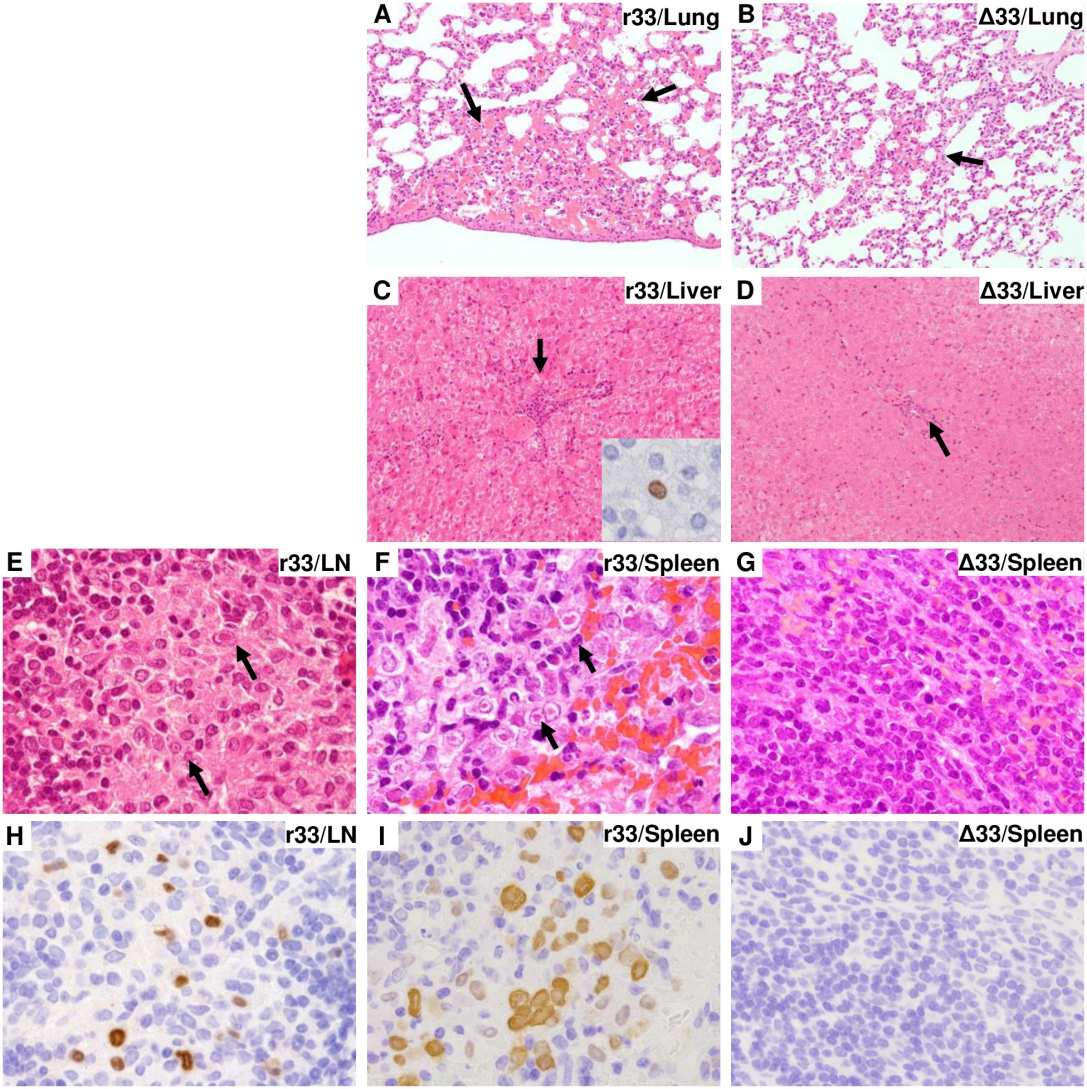
Pathological findings in r33- and Δ33-infected dams at 6 days p.i. Formalin-fixed specimens prepared from the dams described in the legend for Fig 5 were histologically analyzed. **(A, B)** Severe inflammatory cell infiltration with hemorrhage was observed more frequently in the lungs of the r33-infected dams (A) than in those of Δ33-infected dams (B). **(C, D)** Severe inflammation was observed in the portal area in the liver of r33 virus-infected guinea pigs, and GPCMV-antigen-positive cells were found by immunohistochemical analysis (inset) (C). Minor inflammatory infiltration (arrow) was found in the liver of Δ33-infected guinea pigs (D). **(E, H)** HE staining shows intranuclear inclusion bodies in the lymph nodes of one of the r33-infected guinea pigs (E, arrows). Immunohistochemical analysis of cells demonstrated GPCMV-positive cells in the lymph nodes (H). **(F, G, I, J)** Intranuclear inclusion bodies (F, arrows) and many GPCMV-positive cells were found in the spleens of r33-infected guinea pigs (I) by HE staining and by immunohistochemical analysis, respectively. In contrast, no abnormalities or viral antigens were detected in the spleens of Δ33-infected guinea pigs (G, J).

In addition to the higher viral load described above, severe abnormalities were apparent in the lungs, including inflammation and alveolar hemorrhage, of the r33-infected dams (Fig 6A). In addition to the lungs, the livers of the dams infected with r33 showed changes in surface color from red to ochre and in texture from smooth to rough, and one of the livers had a yellowish necrotic lesion (Fig 6B). HE staining analyses demonstrated more frequent infiltration of inflammatory cells into the lungs and livers, especially around the portal regions, of the r33-infected dams (Fig 7A–7D). In addition, immunohistochemical analysis with a monoclonal antibody against a GPCMV early antigen (g-1) detected viral antigens in the liver and lymph nodes of one of the r33-infected dams, although not the dam with the necrotic lesion in the liver (Fig 7C inset and Fig 7H). Importantly, GPCMV antigen-positive cells were detected frequently, especially around white pulps, in the spleens of all 4 dams infected with r33 but in none of the dams infected with Δ33 (Fig 7F, 7G, 7I and 7J). However, the TNF-α mRNA levels in the livers and lungs were similar in r33- and Δ33-infected dams (means ± SEM of threshold cycle numbers after normalization to β-actin for the livers and lungs were r33 = 30.8 ± 0.45 vs. Δ33 = 30.1 ± 0.43 and r33 = 32.9 ± 1.88 vs. Δ33 = 30.6 ± 0.44, respectively), probably because the infected cells represented only a fraction of the total cells in those organs.

### GP33 had no effect on viral growth in dams at 3 weeks p.i

**Fig 8:**
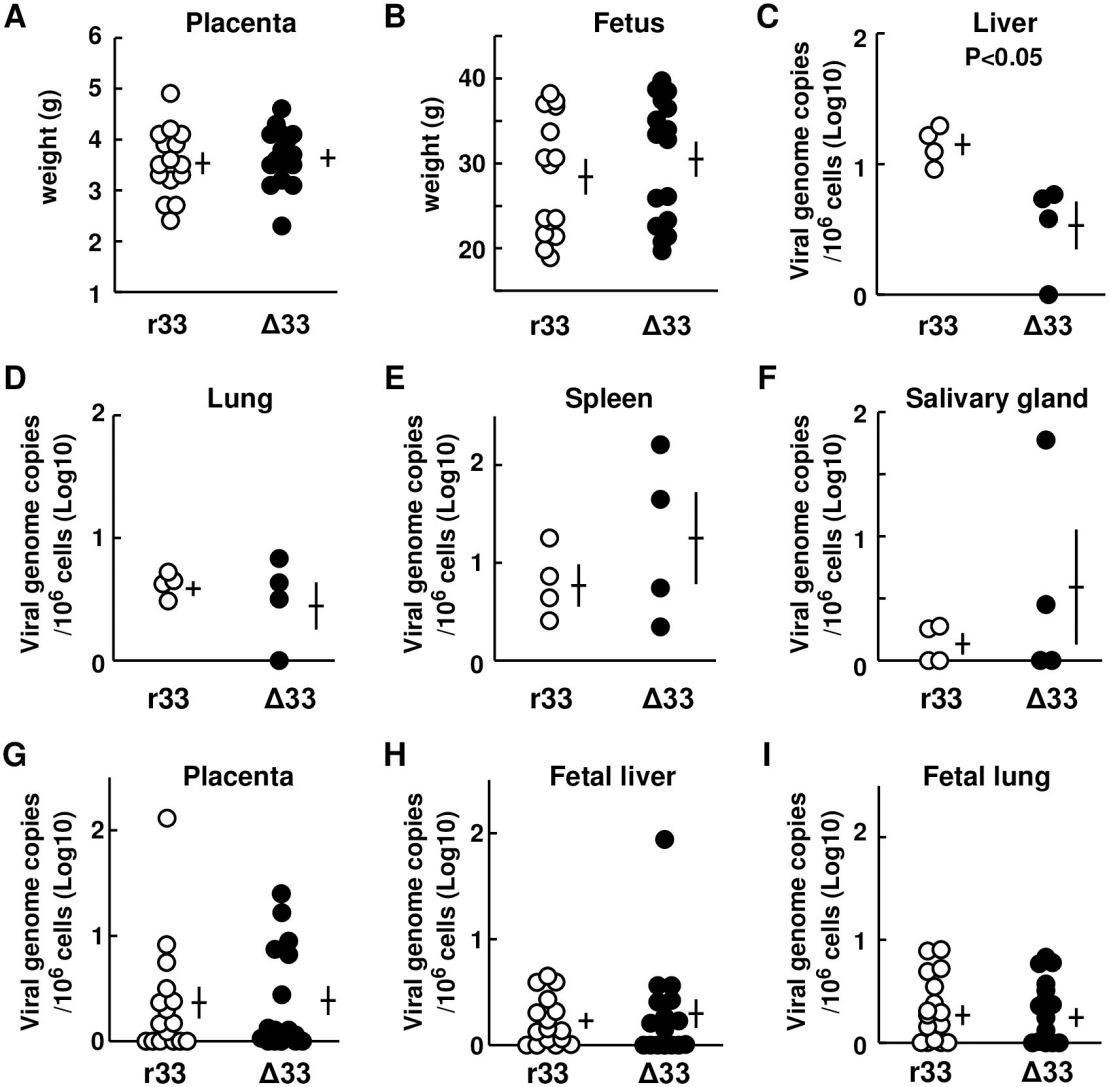
Viral loads in organs at 3 weeks after infection of dams at 4 weeks’ GA. **(A, B)** Pregnant guinea pigs (Hartley) at 4 weeks’ GA were infected s.c. with GPCMV r33 or Δ33 (n = 4 each; 2x10^7^ IUs/animal) and sacrificed at 3 weeks p.i. to collect specimens. Comparison of the weights of placentas (A) and fetuses (B) from dams infected with r33 (open circles) or Δ33 (closed circles). Each circle indicates one placenta or fetus. Means and SEMs of the weights are shown. **(C-F)** GPCMV genome copies per 10^6^ cells of the livers (C), lungs (D), spleens (E), and salivary glands (F) obtained from the dams infected with r33 (open circles) or Δ33 (closed circles). Means and SEMs of genome copies are shown. **(G-I)** GPCMV genome copies per 10^6^ cells of the placentas (G), fetal livers (H) and fetal lungs (I) obtained from fetuses of the dams described above. Means and SEMs of genome copies are shown.

Next, dams (Hartley) at 4 weeks’ GA were infected s.c. with GPCMV r33 or with Δ33 (n = 4 each; 2 x 10^7^ IUs/animal) and sacrificed at 3 weeks p.i. There were no significant differences in the weights of the dams or of their placentas or fetuses (Fig 8A and 8B). In addition, there were no significant differences in viral loads in any of the examined maternal organs (except for the livers, p<0.05), the placentas, or any of the examined fetal organs, between GPCMV r33- and Δ33-infected animals (Fig 8C–8I). As described below, the viral loads in the salivary glands were too small to evaluate the effect of GP33 on viral replication in the salivary glands.

### GP33 was involved in abnormalities in the lungs of dams at 3 weeks p.i

**Fig 9:**
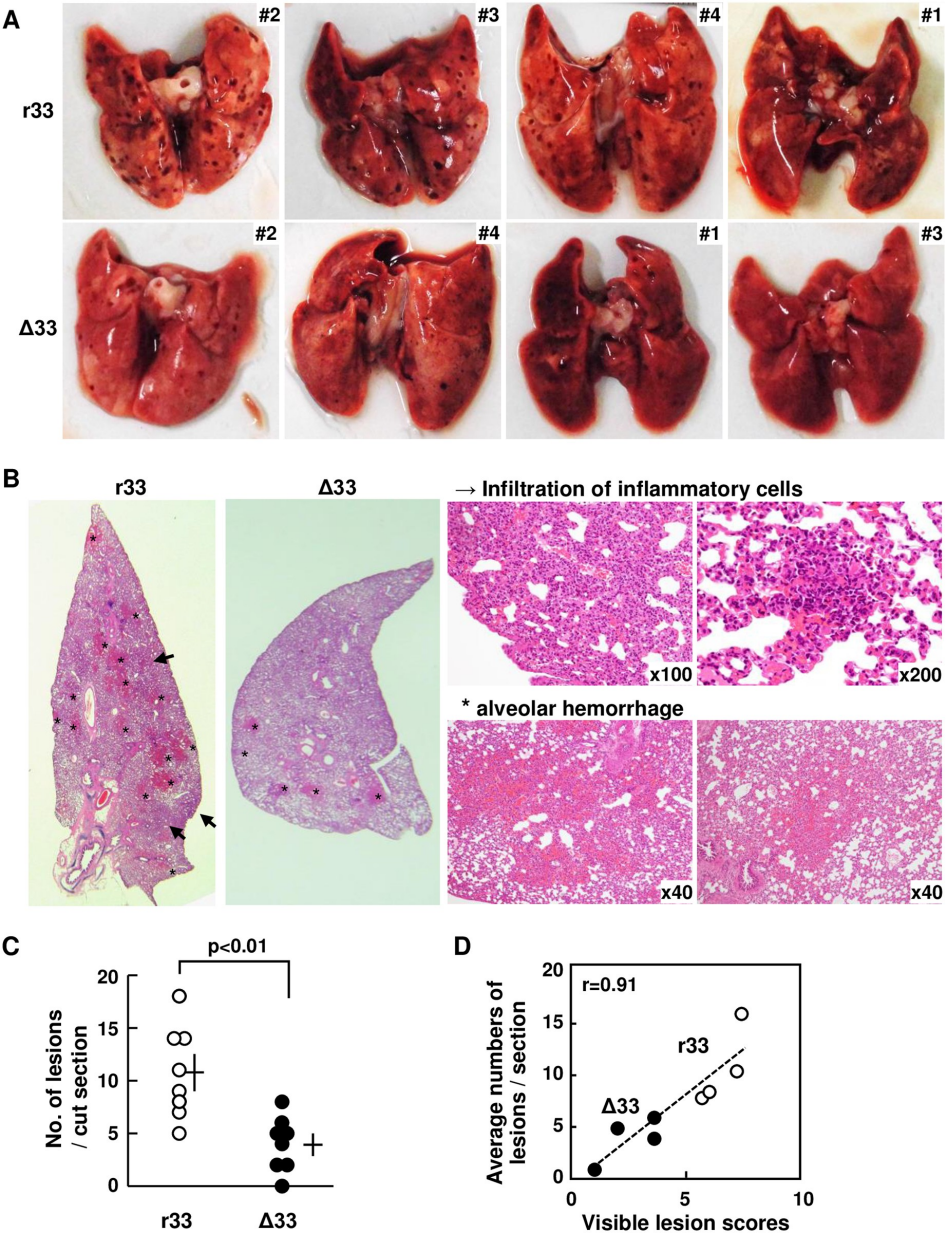
Pathological findings in r33- and Δ33-infected dams at 3 weeks p.i. **(A)** Images of the lungs obtained from the dams infected with r33 (upper row) or Δ33 (lower row), which are described in the legend for Fig 8, are shown in the order of abnormalities in appearance from most severe to mildest. **(B)** Low and high magnifications of the HE staining of sections prepared from the lungs of the dams infected with r33 or Δ33. Asterisks and arrows indicate infiltration of inflammatory cells and alveolar hemorrhage, respectively. **(C)** Average number of lesions observed in two sections prepared from each of the dams infected with r33 (open circles) or Δ33 (closed circles). Means and SEMs are shown. **(D)** Relationship between visible lesion scores on the surface of the lungs determined blindly as described in the Method section and the average number of lesions per cut section. Each circle indicates a lung from each dam.

In spite of the negligible differences in viral loads as shown in Fig 8, more severe abnormalities were apparent in the lungs of the dams infected with r33 than in those infected with Δ33 (Fig 9A). The abnormalities were histologically analyzed, and irrespective of the GP33 status, the lungs of the dams showed infiltration of inflammatory cells and alveolar hemorrhage. However, the number of lesions in the lungs of the dams infected with r33 and with Δ33 were 10.75 ± 1.53 and 4.00 ± 0.91 per section, respectively, and this 2.7-fold difference was statistically significant (p<0.01) (Fig 9B and 9C). In addition, the pathologically obtained number of lesions and the visible lesions on the surface of the lungs scored as described in the Method section showed a good correlation (correlation coefficient r = 0.91, p<0.005) (Fig 9D). Virus antigens were below the detection level by immunohistochemistry with monoclonal anti-GPCMV early antigen (g-1), as viruses are usually cleared from most organs by 3 weeks p.i.

### GPCMV strains derived from our BAC had attenuated phenotypes

**Fig 10:**
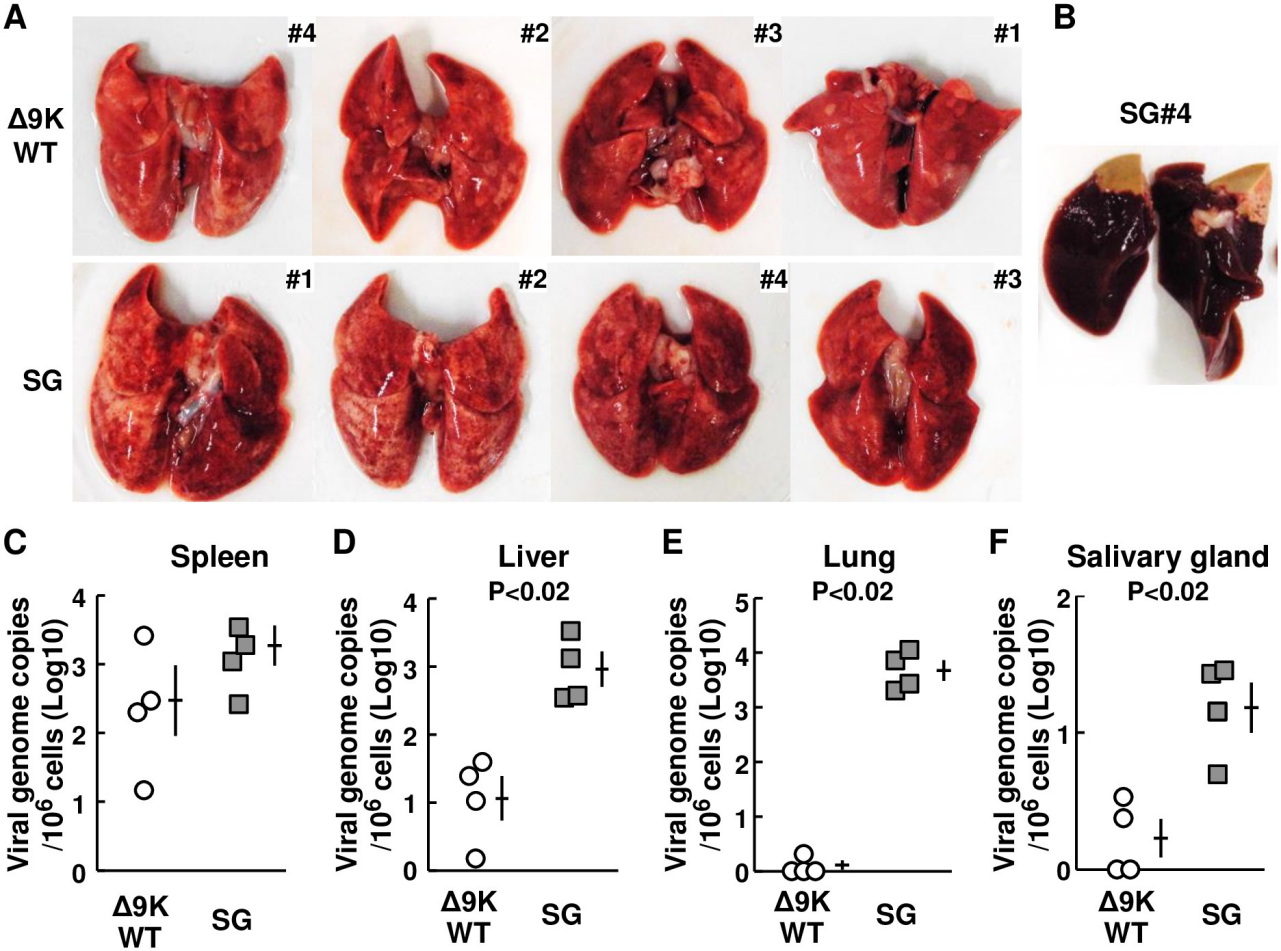
BAC-derived GPCMV strains showed attenuated phenotypes. Pregnant guinea pigs (Hartley) at 4 weeks’ GA were infected s.c. with GPCMVΔ9K WT or with GPCMV SG (n = 4 each; 3x10^6^ IUs/animal) and sacrificed at 6 days p.i. to collect specimens. **(A)** Images of the lungs obtained from the dams infected with GPCMVΔ9K WT (upper row) or GPCMV SG (lower row) are shown in the order of abnormalities in appearance from most severe to mildest. **(B)** An image of the liver with necrotic lesion that was obtained from one of the dams infected with GPCMV SG. **(C-F)** GPCMV genome copies per 10^6^ cells of the spleens (C), livers (D), lungs (E), and salivary glands (F) obtained from the dams infected with GPCMVΔ9K WT (open circles) or GPCMV SG (squares). Means and SEMs are shown.

Schleiss and his colleagues reported that the lack of the GP1 gene encoding a macrophage inflammatory protein 1 (MIP-1) homolog attenuated in vivo phenotypes of GPCMV and that the MIP-1 homolog signals via CCR1 [30,31]. GPCMVΔ9K WT and its derivatives r33 and Δ33 were prepared from pBAC-GPCMVΔ9K that lacks a 9 kb region of the GPCMV genome [32]. At least MIP-1 homolog is encoded in the 9 kb region, although any other ORFs are not identified. To confirm that the lack of the 9 kb region results in the reported attenuated phenotypes and to see whether the lack of the region increases lung and liver pathology of pregnant guinea pigs, dams (Hartley) at 4 weeks’ GA were infected s.c. with GPCMVΔ9K WT lacking the 9 kb region or with GPCMV SG, a strain that contains the 9 kb region and has the full capacity to grow in animals [33] (n = 4 each; 3x10^6^ IUs) and sacrificed at 6 days p.i. As GPCMV SG was more virulent than GPCMVΔ9K WT in preliminary experiments, animals were infected with about one fourth of the dose used in Fig 5. We found that GPCMV SG showed more virulent viral growth phenotype, including more severe inflammatory lesions in their lungs (Fig 10A), a severe large necrotic lesion in one of the livers (Fig 10B), and higher viral loads in the livers, lungs, and salivary glands as compared to GPCMVΔ9K WT (Fig 10D–10F). In addition, in a separate experiment, viral genome copies per 10^6^ cells (Log10) in the salivary glands of dams at 4 weeks’ GA (n = 4) that were infected s.c. with GPCMV SG (3 x 10^6^ IUs/animal) and sacrificed at 3 weeks p.i. were 6.5 ± 0.9, whereas those of dams infected with the BAC-derived strains were negligible as shown in Fig 8. Thus, the lack of the 9 kb region attenuated GPCMV infection significantly but it did not increase lung and liver pathology.

## Discussion

In this study, we determined the cDNA structure of GP33 and demonstrated that GP33 activated the CRE- and NFAT-, but not the NFκB-signal pathway. Phylogenetically, GPCMV lies between MCMV and HCMV [33,34], but in the case of GP33 homologs, it is likely that GP33 is functionally closer to HCMV UL33, as i) GP33 and UL33 contain the DRY motif, whereas M33 and R33 contain the NRY motif, and ii) GP33 and UL33 activate the CRE- and NFAT-, but not the NFκB-signal pathway, whereas M33 and R33 activate all three pathways. Although M33 and R33, but not UL33, induce migration of smooth muscle cells [17,35], it remains unclear whether GP33 behaves in a similar manner to rodent GP33 homologs or to UL33.

GP33 was dispensable for infection both in tissue cultures and in normal animals. In pregnant animals, however, r33 encoding GP33 induced significantly more frequent lesions and higher viral loads in the maternal organs at 6 days p.i. Potential factors affecting the difference in disease pathogenesis between normal and pregnant animals could include i) age of animals, ii) immune responses, iii) chemokine network, and iv) routes of viral inoculation. First, it is unlikely that older animals, in this case, the dams, are more susceptible to GPCMV, as younger animals were more prone to body weight loss after virus challenge [8]. While there is a recent study demonstrating functional impairment of CMV-reactive cellular immunity in pregnant women based on an IFN-γ ELISPOT assay [36], issues surrounding immune responses and susceptibility to infectious diseases during the pregnancy have long been controversial [37]. As the chemokine network in pregnancy significantly contributes to the maintenance of pregnancy, especially to maternal-fetal tolerance and to placentation [38,39], the chemokine profiles specific to the pregnancy may have impact on GP33-mediated pathogenesis. M33 plays an essential role in smooth muscle cell migration in a CCL5-dependent manner, despite its ligand-independent activation of signal pathways in transiently transfection experiments [17]. As an analogy, GP33 could respond to a certain ligand in vivo, such as in the context of pregnancy, in spite of ligand-independent activation shown in this work. Finally, infection via different inoculation routes yields different disease outcomes (reviewed in [40]). For example, CX3CR1 deficiency of mice compromised dissemination of MCMV to the salivary glands from a local footpad inoculation but not from systemic intraperitoneal inoculation [41]. A recent study demonstrated that the lack of M33 hampered viral spread from lymph nodes to the lungs after the i.n. administration of MCMV [22]. One of the limitations of our study is the artificial routes of infection, i.p. for non-pregnant normal animals and s.c. for pregnant animals, while i.n. infection apparently represents the natural infection route.

The most striking phenotype for M33 or R33 mutant viruses is the inability to replicate in the salivary glands [14,19,42,43]. However, we could not evaluate whether GP33 is essential for replication in the salivary glands, as the GPCMV strains used in this study showed significantly reduced viral loads in the salivary glands, probably due to the lack of the MIP-1 homolog. Although the lack of the 9 kb region encoding the MIP-1 homolog resulted attenuated phenotypes, it did not increase lung and liver pathology in pregnant animals. Therefore, it is clear that the deletion of GP33 confers additional attenuation that could be observed in pregnant animals.

The maternal organs of the dams infected with GPCMV r33 showed higher viral loads and active viral replication at 6 days p.i. and more severe inflammation in the lung at 3 weeks p.i., although viral loads in the maternal organs were almost undetectable at 3 weeks p.i. Our hypothetical view would be that GP33 transiently increases viral activities, which triggers inflammation in the maternal visceral organs, but the balance between viral activities and viral clearance by immune responses determines pathological outcomes. Further studies on immune correlates will be required. In addition to the maternal organs, higher viral loads in the placentas of the dams at 6 days p.i. suggest that GP33 has some pathological roles in congenital diseases. However, due to the small size of the infected fetuses, we could not obtain organs from a sufficient number of fetuses to analyze fetal transmission at 6 days p.i. In addition, the lack of the difference in the weights of the placenta and fetus as well as in viral loads of any of the examined fetal organs, between r33- and Δ33-infected dams at 3 weeks p.i., makes it hard to conclude that GP33 is involved in fetal transmission. Not only direct infection of fetuses causes congenital diseases but also infection of the placenta may impair the normal growth of fetuses indirectly, for example, by CMV-induced cytokine modulation [25,44]. Also vascular insufficiency due to CMV-mediated injury of endothelial cells could compromise perfusion to the developing fetus or to the placenta, resulting in hypoxia and growth restriction [45,46]. Therefore, it would be essential to analyze pup survival and pup viral loads to see whether GP33 contributes to any of the pathogenic aspects of congenital infection.

The importance of our study lies in the demonstration of GP33 involvement in the pathological effects in some organs. It is intriguing that GP33 appears to enhance the accumulation of GPCMV-infected cells in the spleen. We hypothesize that an increase of inflammatory cytokines by GP33 allows the infection and migration of monocytes/macrophages in the spleen to the organs for viral dissemination as well as for the induction of pathological lesions. Alternatively, GP33 may facilitate transient inflammation that is induced by the chemokine network specific to the pregnancy. Expression of GP33, such as by transduction with a recombinant adenovirus vector, in the absence of GPCMV infection or in the presence of Δ33 infection might help the understanding of the observed phenomena. In addition to GP33, GPCMV encodes GP78, the UL78 homolog. It would be interesting to see whether the presence of GP78 compensates for the GP33 defect and whether GP33 forms a heterodimer with GP78 or other GPCR homologs for unexpected functions. Although our study had some limitations, our observations regarding the pathogenic role of GP33 during pregnancy warrants further studies on the functions of GPCMV vGPCRs.

In conclusion, GP33, a GPCR encoded by GPCMV, contributes to increasing viral activities transiently in the maternal spleens and livers and in the placentas as well as to inducing inflammation in some maternal organs during pregnancy.

## Materials and methods

### RACE and construction of GP33-expressing plasmids

GPL (CCL-158, ATCC USA) were cultured in F-12 medium supplemented with 10% fetal bovine serum (FBS, HyClone, USA) and infected with GPCMV. Two days later, RNA samples were extracted from the infected cells with RNeasy Mini Kit (Qiagen). The cDNA was synthesized from the extracted RNA with SMARTer RACE 5´/3´Kit (Clontech). The obtained PCR products were purified using a DNA fragment purification kit (MagExtractor, TOYOBO, Japan), and their nucleotide sequences were determined. The UL33 ORF prepared from HCMV-infected fibroblasts, the ORF predicted initially as GP33 (GP33s), the GP33 ORF in the cDNA obtained by the RACE analyses, and the GP33 ORF with FLAG-tag at the carboxyl end were cloned into pcDNA3.0 (Invitrogen), resulting in pcDNA-UL33, -GP33s, -GP33 and -GP33F, respectively. The GP33 ORF was also cloned between EcoRI and BamHI sites of pEGFP-N1 (Clontech), resulting in pEGFP-GP33 to express GP33-EGFP fusion protein. Cells transfected with pEGFP-GP33 were fixed with paraformaldehyde and observed under a confocal microscope.

### Transient transfection assay

COS-7 cells (product no. CRL1651, ATCC) in 96-well plates were cultured in Dulbecco’s modified medium supplemented with 10% FBS and penicillin/streptomycin, and transiently transfected with a reporter plasmid [pCRE-luc, pNFAT-luc, or pNFκB-luc (Clontech)], an internal control plasmid (pRL-EF1α) [47], and an effector plasmid (pcDNA-GP33s and -GP33) or positive control plasmids, by adding 200-fold diluted lipofectamine 2000 (Invitrogen) and cultured for 6 h. Total amount of DNA for each condition was adjusted by adding the empty vector. The cells were then cultured in a serum-free medium for 24 h and rinsed with PBS. The cells were treated with a Dual-luciferase reporter assay kit (Promega), and the luciferase activities of the cells were measured by GloMax (Promega). pF4A-CMV-GαqQ209L and a plasmid expressing FLAG-MyD88, kindly provided by H. Ueda (Gifu Univ.) and A. Takaoka (Hokkaido Univ.), respectively, were used as positive control plasmids for the NFAT- and NFκB-pathways, respectively.

### Construction of GP33 mutants

pBAC-GPCMVΔ9K, a BAC that contains a GFP expression cassette and the GPCMV genome lacking an 8.9-kb sequence [32], was used for the generation of GP33 mutants as follows. GPCMV with a knock-out mutation in the GP33 gene and that with a wild-type sequence were prepared from the same backbone as follows. First, the rpsL-neo cassette was amplified by PCR using primers GP33rpsL-F and GP33rpsL-R (S1 Table) that add a 50-bp GPCMV sequence flanking the GP33 ORF and used for recombination with the GPCMV region 54,807–56,090 (the numbers are based on Genbank acc. no. AB592928;[33]) of pBAC-GPCMVΔ9K using the RedET recombination system (GeneBridges), resulting in pBAC-GPCMVΔ9K::rpsL-neo/GP33. A fragment containing the GPCMV region 54,698–56181 was amplified by PCR with primers GP33BS-F and -R, and cloned into the BamHI and EcoRI sites of pBluescriptII KS(+). Next, the resultant plasmid was mutagenized using a commercial kit (QuickChange Lighting, Agilent) with the primers GP33KO-F and -R (S1 Table) to introduce a “TGAT” sequence, which inserts a stop-codon mutation at amino acid position 30, accompanied by a frame-shift mutation, into the GP33 ORF (Fig 1A). The rpsL-neo cassette in pBAC-GPCMVΔ9K::rpsL-neo/GP33 was replaced with the GPCMV sequences containing the mutated “TGAT” or wild-type sequence by electroporation of the PCR fragments (54,698–56,179) amplified from the plasmids using primers GP33-F and -R (S1 Table), resulting in pBAC-GPCMVΔ9K-r33 and -Δ33.

GPCMV WT, r33 and Δ33, were recovered by transfection of BAC DNAs, the parental pBAC-GPCMVΔ9K and the manipulated pBAC-GPCMVΔ9K-r33 and -Δ33, respectively, into GPL cells. After observation of plaque formation, the recovered viruses were amplified a couple of times by the co-culturing of infected cells with uninfected cells. Finally, cell-free virus stocks were prepared and concentrated by ultracentrifugation (20,000 *g* for 2 h) in a 20% sucrose step gradient as described previously [32]. RFLP analyses of the GPCMV-BACs with EcoRI and HindIII demonstrated no apparent differences in the genome structures among GPCMV WT, r33 and Δ33. In addition, the integrity of the locus encoding GP129, GP131, and GP133 was confirmed. As the viral stocks prepared from the two BAC clones for each strain behaved similarly in the following experiments, the results obtained from only one of them are shown. Titers of the GPCMV-BAC stocks were determined by counting GFP-positive foci after infection of GPL cells. Although the infectivity depended on cell type, we used those titers to calculate multiplicities of infection (MOIs).

### Animal experiments

Four- or 5-week-old female normal guinea pigs (strain Hartley) and pregnant guinea pigs at 4 weeks’ GA were all purchased from a commercial breeder (SLC Inc., Hamamatsu, Japan). Since the company allows mating for two weeks, 4 weeks’ GA means 4 ± 1 weeks’ GA. Animals were randomly grouped based on the body weights. Blood specimens were drawn from ear veins using heparinized micro-hematocrit capillaries (Harschmann, Germany) for serological tests. All tested animals were seronegative for GPCMV. Normal and pregnant animals were infected with GPCMV i.p. and s.c., respectively. Animals were euthanized at the indicated days p.i. The visible lesions on the surface of the lungs were scored blindly from “1” to “10” based on numbers and sizes of lesions, by 5 lab members who worked in the bacteriology field and were not involved in this study.

### Infection of guinea pig epithelial cells and splenic macrophages

Guinea pig epithelial cell clone GPE-7 was established by immortalization of primary renal cells obtained from guinea pigs with SV40 T antigen as described previously [48].

Splenic monocytes were prepared and treated with 100 nM phorbol 12-myristate 13-acetate (PMA) for 2 days to obtain macrophages as described previously [32].

### Real-time RT-PCR assay for TNF-α

Total RNA samples were prepared from infected GPL cells at 24 hrs p.i. or from guinea pig tissues using a commercial kit (RNeasy Plus Mini kit, Qiagen), and reverse-transcribed using PrimeScript RT master mix (Takara Bio). Real-time PCR of the reverse transcribed products was performed using a commercial kit (GoTaq qPCR Master Mix, Promega) with primers amplifying a region containing an exon junction of the guinea pig TNF-α or β-actin gene (S1 Table).

### Quantitative PCR

Total DNA samples were prepared using Maxwell 16 Tissue DNA purification kit (Promega), and the quantification of cellular β-actin and GPCMV GP83 genes was performed as described previously [8].

### Histology

All organs obtained from sacrificed animals were fixed in 10% buffered formalin. Formalin-fixed specimens were embedded in paraffin, sectioned, and stained with hematoxylin and eosin (HE), as described previously [8]. Serial sections were used for HE staining and immunohistochemistry. Immunohistochemical analysis was performed using the monoclonal antibody g-1, which detects a GPCMV early antigen [5], as a primary antibody. For the second- and third-phase immunostaining reagents, a biotinylated F(ab)^2^ fragment of anti-mouse immunoglobulin (DAKO) and peroxidase-conjugated streptavidin (DAKO) were used. DAB was used as a chromogen, and the slides were counterstained with hematoxylin.

### Statistical analyses

Statistical analyses were performed using GraphPad Prism ver.7 (GraphPad Software, Inc., CA, US). The statistical significances of differences in the weights and viral loads between the two groups were calculated using the Mann-Whitney U test, with values of p<0.05 considered significant. The correlation between the two types of lesion scores was evaluated by Pearson correlation coefficient (r).

### Ethics statement

All animal procedures, including euthanasia using CO_2_, described in this study were approved by the Animal Care and Use Committee of Gifu Pharmaceutical University (approval no. 2016–132, -220, -320, 2017–021, -079, -214, 2018–117). The same procedures were also approved by the Animal Care and Use Committee of Gifu University (approval no. 28–57, -75, -96, 29–37, -49, -91, 30–94), as we used a P2 animal facility in the Life Science Research Center of Gifu University for infection of guinea pigs with GPCMV. All of the above mentioned animal procedures are in accordance with the following Japanese laws and guidelines: “Action on Welfare and Management of Animals”(law) and “Fundamental Guidelines for Proper Conduct of Animal Experiment and Related Activities in Academic Research Institutions under the jurisdiction of the Ministry of Education, Culture, Sports, Science and Technology”(guidelines), “Act on the Conservation and Sustainable Use of Biological Diversity through Regulations on the Use of Living Modified Organisms”(law), and “Act on the Prevention of Infectious Diseases and Medical Care for Patients with Infectious Diseases”(law).

## Supporting information

S1 Table Primers used in this study. (DOCX)
