# Supplementary material for: Roles of GP33, a guinea pig cytomegalovirus-encoded G protein-coupled receptor homolog, in cellular signaling, viral growth and inflammation in vitro and in vivo
Source: PLoS Pathog. 2018 Dec 20;14(12):e1007487. doi: 10.1371/journal.ppat.1007487 (PMC6319746; doi:10.1371/journal.ppat.1007487)
Supplement: S1 Table — (DOCX) [file ppat.1007487.s001.docx]

**Supporting information**

**S1 Table. Primers used in this study.**
